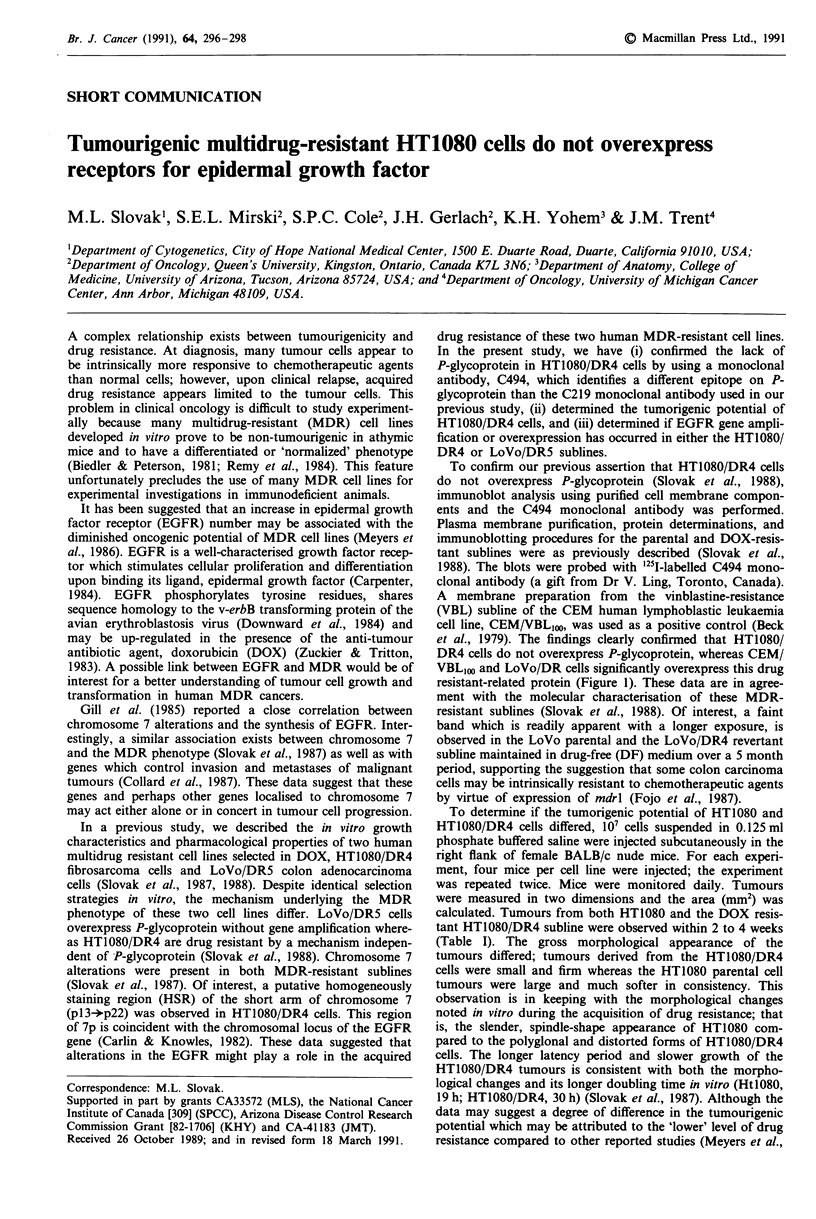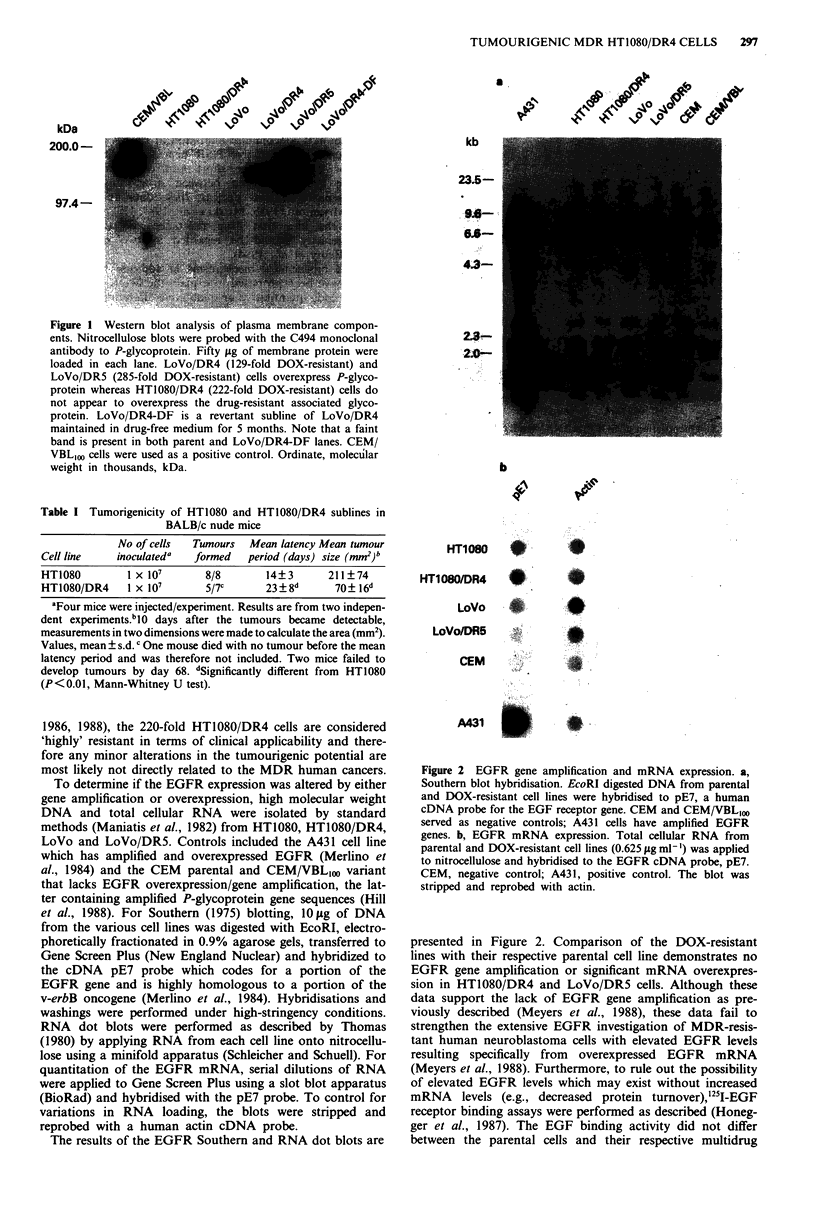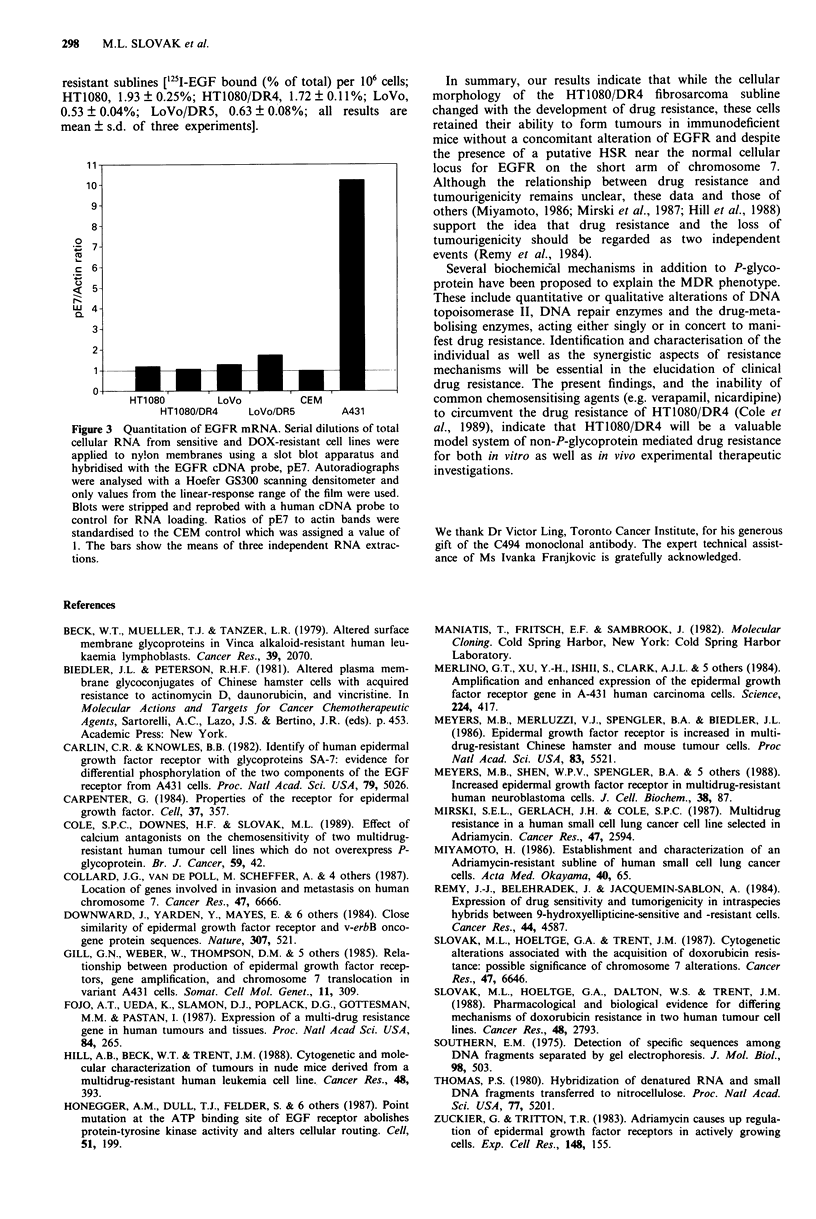# Tumourigenic multidrug-resistant HT1080 cells do not overexpress receptors for epidermal growth factor.

**DOI:** 10.1038/bjc.1991.295

**Published:** 1991-08

**Authors:** M. L. Slovak, S. E. Mirski, S. P. Cole, J. H. Gerlach, K. H. Yohem, J. M. Trent

**Affiliations:** Department of Cytogenetics, City of Hope National Medical Center, Duarte, California 91010.

## Abstract

**Images:**


Br. J. Cancer (1991), 64, 296-298 © Macmillan Press Ltd., 1991

SHORT COMMUNICATION

### Tumourigenic multidrug-resistant HT1080 cells do not overexpress receptors for epidermal growth factor

M.L. Slovak[1], S.E.L. Mirski[2], S.P.C. Cole[2], J.H. Gerlach[2], K.H. Yohem[3] & J.M. Trent[4]

[1]*Department of Cytogenetics, City of Hope National Medical Center, 1500 E. Duarte Road, Duarte, California 91010, USA;* [2]*Department of Oncology, Queen's University, Kingston, Ontario, Canada K7L 3N6;* [3]*Department of Anatomy, College of Medicine, University of Arizona, Tucson, Arizona 85724, USA; and* [4]*Department of Oncology, University of Michigan Cancer Center, Ann Arbor, Michigan 48109, USA.*

A complex relationship exists between tumourigenicity and drug resistance. At diagnosis, many tumour cells appear to be intrinsically more responsive to chemotherapeutic agents than normal cells; however, upon clinical relapse, acquired drug resistance appears limited to the tumour cells. This problem in clinical oncology is difficult to study experimentally because many multidrug-resistant (MDR) cell lines developed *in vitro* prove to be non-tumourigenic in athymic mice and to have a differentiated or 'normalized' phenotype (Biedler & Peterson, 1981; Remy *et al.*, 1984). This feature unfortunately precludes the use of many MDR cell lines for experimental investigations in immunodeficient animals.

It has been suggested that an increase in epidermal growth factor receptor (EGFR) number may be associated with the diminished oncogenic potential of MDR cell lines (Meyers *et al.*, 1986). EGFR is a well-characterised growth factor receptor which stimulates cellular proliferation and differentiation upon binding its ligand, epidermal growth factor (Carpenter, 1984). EGFR phosphorylates tyrosine residues, shares sequence homology to the v-*erb*B transforming protein of the avian erythroblastosis virus (Downward *et al.*, 1984) and may be up-regulated in the presence of the anti-tumour antibiotic agent, doxorubicin (DOX) (Zuckier & Tritton, 1983). A possible link between EGFR and MDR would be of interest for a better understanding of tumour cell growth and transformation in human MDR cancers.

Gill *et al.* (1985) reported a close correlation between chromosome 7 alterations and the synthesis of EGFR. Interestingly, a similar association exists between chromosome 7 and the MDR phenotype (Slovak *et al.*, 1987) as well as with genes which control invasion and metastases of malignant tumours (Collard *et al.*, 1987). These data suggest that these genes and perhaps other genes localised to chromosome 7 may act either alone or in concert in tumour cell progression.

In a previous study, we described the *in vitro* growth characteristics and pharmacological properties of two human multidrug resistant cell lines selected in DOX, HT1080/DR4 fibrosarcoma cells and LoVo/DR5 colon adenocarcinoma cells (Slovak *et al.*, 1987, 1988). Despite identical selection strategies *in vitro*, the mechanism underlying the MDR phenotype of these two cell lines differ. LoVo/DR5 cells overexpress *P*-glycoprotein without gene amplification whereas HT1080/DR4 are drug resistant by a mechanism independent of *P*-glycoprotein (Slovak *et al.*, 1988). Chromosome 7 alterations were present in both MDR-resistant sublines (Slovak *et al.*, 1987). Of interest, a putative homogeneously staining region (HSR) of the short arm of chromosome 7 (p13→p22) was observed in HT1080/DR4 cells. This region of 7p is coincident with the chromosomal locus of the EGFR gene (Carlin & Knowles, 1982). These data suggested that alterations in the EGFR might play a role in the acquired drug resistance of these two human MDR-resistant cell lines. In the present study, we have (i) confirmed the lack of *P*-glycoprotein in HT1080/DR4 cells by using a monoclonal antibody, C494, which identifies a different epitope on *P*-glycoprotein than the C219 monoclonal antibody used in our previous study, (ii) determined the tumorigenic potential of HT1080/DR4 cells, and (iii) determined if EGFR gene amplification or overexpression has occurred in either the HT1080/DR4 or LoVo/DR5 sublines.

To confirm our previous assertion that HT1080/DR4 cells do not overexpress *P*-glycoprotein (Slovak *et al.*, 1988), immunoblot analysis using purified cell membrane components and the C494 monoclonal antibody was performed. Plasma membrane purification, protein determinations, and immunoblotting procedures for the parental and DOX-resistant sublines were as previously described (Slovak *et al.*, 1988). The blots were probed with $^{125}$I-labelled C494 monoclonal antibody (a gift from Dr V. Ling, Toronto, Canada). A membrane preparation from the vinblastine-resistance (VBL) subline of the CEM human lymphoblastic leukaemia cell line, CEM/VBL$_{100}$, was used as a positive control (Beck *et al.*, 1979). The findings clearly confirmed that HT1080/DR4 cells do not overexpress *P*-glycoprotein, whereas CEM/VBL$_{100}$ and LoVo/DR cells significantly overexpress this drug resistant-related protein (Figure 1). These data are in agreement with the molecular characterisation of these MDR-resistant sublines (Slovak *et al.*, 1988). Of interest, a faint band which is readily apparent with a longer exposure, is observed in the LoVo parental and the LoVo/DR4 revertant subline maintained in drug-free (DF) medium over a 5 month period, supporting the suggestion that some colon carcinoma cells may be intrinsically resistant to chemotherapeutic agents by virtue of expression of *mdr*1 (Fojo *et al.*, 1987).

To determine if the tumorigenic potential of HT1080 and HT1080/DR4 cells differed, $10^7$ cells suspended in 0.125 ml phosphate buffered saline were injected subcutaneously in the right flank of female BALB/c nude mice. For each experiment, four mice per cell line were injected; the experiment was repeated twice. Mice were monitored daily. Tumours were measured in two dimensions and the area (mm$^2$) was calculated. Tumours from both HT1080 and the DOX resistant HT1080/DR4 subline were observed within 2 to 4 weeks (Table I). The gross morphological appearance of the tumours differed; tumours derived from the HT1080/DR4 cells were small and firm whereas the HT1080 parental cell tumours were large and much softer in consistency. This observation is in keeping with the morphological changes noted *in vitro* during the acquisition of drug resistance; that is, the slender, spindle-shape appearance of HT1080 compared to the polyglonal and distorted forms of HT1080/DR4 cells. The longer latency period and slower growth of the HT1080/DR4 tumours is consistent with both the morphological changes and its longer doubling time *in vitro* (Ht1080, 19 h; HT1080/DR4, 30 h) (Slovak *et al.*, 1987). Although the data may suggest a degree of difference in the tumourigenic potential which may be attributed to the 'lower' level of drug resistance compared to other reported studies (Meyers *et al.*,

Correspondence: M.L. Slovak.
Supported in part by grants CA33572 (MLS), the National Cancer Institute of Canada [309] (SPCC), Arizona Disease Control Research Commission Grant [82-1706] (KHY) and CA-41183 (JMT).
Received 26 October 1989; and in revised form 18 March 1991.

**Figure 1** Western blot analysis of plasma membrane components. Nitrocellulose blots were probed with the C494 monoclonal antibody to *P*-glycoprotein. Fifty μg of membrane protein were loaded in each lane. LoVo/DR4 (129-fold DOX-resistant) and LoVo/DR5 (285-fold DOX-resistant) cells overexpress *P*-glycoprotein whereas HT1080/DR4 (222-fold DOX-resistant) cells do not appear to overexpress the drug-resistant associated glycoprotein. LoVo/DR4-DF is a revertant subline of LoVo/DR4 maintained in drug-free medium for 5 months. Note that a faint band is present in both parent and LoVo/DR4-DF lanes. CEM/$VBL_{100}$ cells were used as a positive control. Ordinate, molecular weight in thousands, kDa.

**Table I** Tumorigenicity of HT1080 and HT1080/DR4 sublines in BALB/c nude mice

| *Cell line* | *No of cells inoculated*[a] | *Tumours formed* | *Mean latency period (days)* | *Mean tumour size* ($mm^2$)[b] |
|---|---|---|---|---|
| HT1080 | $1 \times 10^7$ | 8/8 | 14±3 | 211±74 |
| HT1080/DR4 | $1 \times 10^7$ | 5/7[c] | 23±8[d] | 70±16[d] |

[a]Four mice were injected/experiment. Results are from two independent experiments.[b]10 days after the tumours became detectable, measurements in two dimensions were made to calculate the area ($mm^2$). Values, mean ± s.d. [c] One mouse died with no tumour before the mean latency period and was therefore not included. Two mice failed to develop tumours by day 68. [d]Significantly different from HT1080 ($P<0.01$, Mann-Whitney U test).

1986, 1988), the 220-fold HT1080/DR4 cells are considered 'highly' resistant in terms of clinical applicability and therefore any minor alterations in the tumourigenic potential are most likely not directly related to the MDR human cancers.

To determine if the EGFR expression was altered by either gene amplification or overexpression, high molecular weight DNA and total cellular RNA were isolated by standard methods (Maniatis *et al.*, 1982) from HT1080, HT1080/DR4, LoVo and LoVo/DR5. Controls included the A431 cell line which has amplified and overexpressed EGFR (Merlino *et al.*, 1984) and the CEM parental and CEM/$VBL_{100}$ variant that lacks EGFR overexpression/gene amplification, the latter containing amplified *P*-glycoprotein gene sequences (Hill *et al.*, 1988). For Southern (1975) blotting, 10 μg of DNA from the various cell lines was digested with EcoRI, electrophoretically fractionated in 0.9% agarose gels, transferred to Gene Screen Plus (New England Nuclear) and hybridized to the cDNA pE7 probe which codes for a portion of the EGFR gene and is highly homologous to a portion of the v-*erb*B oncogene (Merlino *et al.*, 1984). Hybridisations and washings were performed under high-stringency conditions. RNA dot blots were performed as described by Thomas (1980) by applying RNA from each cell line onto nitrocellulose using a minifold apparatus (Schleicher and Schuell). For quantitation of the EGFR mRNA, serial dilutions of RNA were applied to Gene Screen Plus using a slot blot apparatus (BioRad) and hybridised with the pE7 probe. To control for variations in RNA loading, the blots were stripped and reprobed with a human actin cDNA probe.

The results of the EGFR Southern and RNA dot blots are

**Figure 2** EGFR gene amplification and mRNA expression. **a**, Southern blot hybridisation. *Eco*RI digested DNA from parental and DOX-resistant cell lines were hybridised to pE7, a human cDNA probe for the EGF receptor gene. CEM and CEM/$VBL_{100}$ served as negative controls; A431 cells have amplified EGFR genes. **b**, EGFR mRNA expression. Total cellular RNA from parental and DOX-resistant cell lines (0.625 μg $ml^{-1}$) was applied to nitrocellulose and hybridised to the EGFR cDNA probe, pE7. CEM, negative control; A431, positive control. The blot was stripped and reprobed with actin.

presented in Figure 2. Comparison of the DOX-resistant lines with their respective parental cell line demonstrates no EGFR gene amplification or significant mRNA overexpression in HT1080/DR4 and LoVo/DR5 cells. Although these data support the lack of EGFR gene amplification as previously described (Meyers *et al.*, 1988), these data fail to strengthen the extensive EGFR investigation of MDR-resistant human neuroblastoma cells with elevated EGFR levels resulting specifically from overexpressed EGFR mRNA (Meyers *et al.*, 1988). Furthermore, to rule out the possibility of elevated EGFR levels which may exist without increased mRNA levels (e.g., decreased protein turnover),$^{125}$I-EGF receptor binding assays were performed as described (Honegger *et al.*, 1987). The EGF binding activity did not differ between the parental cells and their respective multidrug resistant sublines [$^{125}$I-EGF bound (% of total) per $10^6$ cells; HT1080, 1.93 ± 0.25%; HT1080/DR4, 1.72 ± 0.11%; LoVo, 0.53 ± 0.04%; LoVo/DR5, 0.63 ± 0.08%; all results are mean ± s.d. of three experiments].

**Figure 3** Quantitation of EGFR mRNA. Serial dilutions of total cellular RNA from sensitive and DOX-resistant cell lines were applied to nylon membranes using a slot blot apparatus and hybridised with the EGFR cDNA probe, pE7. Autoradiographs were analysed with a Hoefer GS300 scanning densitometer and only values from the linear-response range of the film were used. Blots were stripped and reprobed with a human cDNA probe to control for RNA loading. Ratios of pE7 to actin bands were standardised to the CEM control which was assigned a value of 1. The bars show the means of three independent RNA extractions.

In summary, our results indicate that while the cellular morphology of the HT1080/DR4 fibrosarcoma subline changed with the development of drug resistance, these cells retained their ability to form tumours in immunodeficient mice without a concomitant alteration of EGFR and despite the presence of a putative HSR near the normal cellular locus for EGFR on the short arm of chromosome 7. Although the relationship between drug resistance and tumourigenicity remains unclear, these data and those of others (Miyamoto, 1986; Mirski *et al.*, 1987; Hill *et al.*, 1988) support the idea that drug resistance and the loss of tumourigenicity should be regarded as two independent events (Remy *et al.*, 1984).

Several biochemical mechanisms in addition to *P*-glycoprotein have been proposed to explain the MDR phenotype. These include quantitative or qualitative alterations of DNA topoisomerase II, DNA repair enzymes and the drug-metabolising enzymes, acting either singly or in concert to manifest drug resistance. Identification and characterisation of the individual as well as the synergistic aspects of resistance mechanisms will be essential in the elucidation of clinical drug resistance. The present findings, and the inability of common chemosensitising agents (e.g. verapamil, nicardipine) to circumvent the drug resistance of HT1080/DR4 (Cole *et al.*, 1989), indicate that HT1080/DR4 will be a valuable model system of non-*P*-glycoprotein mediated drug resistance for both *in vitro* as well as *in vivo* experimental therapeutic investigations.

We thank Dr Victor Ling, Toronto Cancer Institute, for his generous gift of the C494 monoclonal antibody. The expert technical assistance of Ms Ivanka Franjkovic is gratefully acknowledged.

### References

BECK, W.T., MUELLER, T.J. & TANZER, L.R. (1979). Altered surface membrane glycoproteins in Vinca alkaloid-resistant human leukaemia lymphoblasts. *Cancer Res.*, **39**, 2070.

BIEDLER, J.L. & PETERSON, R.H.F. (1981). Altered plasma membrane glycoconjugates of Chinese hamster cells with acquired resistance to actinomycin D, daunorubicin, and vincristine. In *Molecular Actions and Targets for Cancer Chemotherapeutic Agents*, Sartorelli, A.C., Lazo, J.S. & Bertino, J.R. (eds). p. 453. Academic Press: New York.

CARLIN, C.R. & KNOWLES, B.B. (1982). Identify of human epidermal growth factor receptor with glycoproteins SA-7: evidence for differential phosphorylation of the two components of the EGF receptor from A431 cells. *Proc. Natl Acad. Sci. USA*, **79**, 5026.

CARPENTER, G. (1984). Properties of the receptor for epidermal growth factor. *Cell*, **37**, 357.

COLE, S.P.C., DOWNES, H.F. & SLOVAK, M.L. (1989). Effect of calcium antagonists on the chemosensitivity of two multidrug-resistant human tumour cell lines which do not overexpress *P*-glycoprotein. *Br. J. Cancer*, **59**, 42.

COLLARD, J.G., VAN DE POLL, M. SCHEFFER, A. & 4 others (1987). Location of genes involved in invasion and metastasis on human chromosome 7. *Cancer Res.*, **47**, 6666.

DOWNWARD, J., YARDEN, Y., MAYES, E. & 6 others (1984). Close similarity of epidermal growth factor receptor and v-*erb*B oncogene protein sequences. *Nature*, **307**, 521.

GILL, G.N., WEBER, W., THOMPSON, D.M. & 5 others (1985). Relationship between production of epidermal growth factor receptors, gene amplification, and chromosome 7 translocation in variant A431 cells. *Somat. Cell Mol. Genet.*, **11**, 309.

FOJO, A.T., UEDA, K., SLAMON, D.J., POPLACK, D.G., GOTTESMAN, M.M. & PASTAN, I. (1987). Expression of a multi-drug resistance gene in human tumours and tissues. *Proc. Natl Acad Sci. USA*, **84**, 265.

HILL, A.B., BECK, W.T. & TRENT, J.M. (1988). Cytogenetic and molecular characterization of tumours in nude mice derived from a multidrug-resistant human leukemia cell line. *Cancer Res.*, **48**, 393.

HONEGGER, A.M., DULL, T.J., FELDER, S. & 6 others (1987). Point mutation at the ATP binding site of EGF receptor abolishes protein-tyrosine kinase activity and alters cellular routing. *Cell*, **51**, 199.

MANIATIS, T., FRITSCH, E.F. & SAMBROOK, J. (1982). *Molecular Cloning*. Cold Spring Harbor, New York: Cold Spring Harbor Laboratory.

MERLINO, G.T., XU, Y.-H., ISHII, S., CLARK, A.J.L. & 5 others (1984). Amplification and enhanced expression of the epidermal growth factor receptor gene in A-431 human carcinoma cells. *Science*, **224**, 417.

MEYERS, M.B., MERLUZZI, V.J., SPENGLER, B.A. & BIEDLER, J.L. (1986). Epidermal growth factor receptor is increased in multidrug-resistant Chinese hamster and mouse tumour cells. *Proc Natl Acad. Sci. USA*, **83**, 5521.

MEYERS, M.B., SHEN, W.P.V., SPENGLER, B.A. & 5 others (1988). Increased epidermal growth factor receptor in multidrug-resistant human neuroblastoma cells. *J. Cell. Biochem.*, **38**, 87.

MIRSKI, S.E.L., GERLACH, J.H. & COLE, S.P.C. (1987). Multidrug resistance in a human small cell lung cancer cell line selected in Adriamycin. *Cancer Res.*, **47**, 2594.

MIYAMOTO, H. (1986). Establishment and characterization of an Adriamycin-resistant subline of human small cell lung cancer cells. *Acta Med. Okayama*, **40**, 65.

REMY, J.-J., BELEHRADEK, J. & JACQUEMIN-SABLON, A. (1984). Expression of drug sensitivity and tumorigenicity in intraspecies hybrids between 9-hydroxyellipticine-sensitive and -resistant cells. *Cancer Res.*, **44**, 4587.

SLOVAK, M.L., HOELTGE, G.A. & TRENT, J.M. (1987). Cytogenetic alterations associated with the acquisition of doxorubicin resistance: possible significance of chromosome 7 alterations. *Cancer Res.*, **47**, 6646.

SLOVAK, M.L., HOELTGE, G.A., DALTON, W.S. & TRENT, J.M. (1988). Pharmacological and biological evidence for differing mechanisms of doxorubicin resistance in two human tumour cell lines. *Cancer Res.*, **48**, 2793.

SOUTHERN, E.M. (1975). Detection of specific sequences among DNA fragments separated by gel electrophoresis. *J. Mol. Biol.*, **98**, 503.

THOMAS, P.S. (1980). Hybridization of denatured RNA and small DNA fragments transferred to nitrocellulose. *Proc. Natl Acad. Sci. USA*, **77**, 5201.

ZUCKIER, G. & TRITTON, T.R. (1983). Adriamycin causes up regulation of epidermal growth factor receptors in actively growing cells. *Exp. Cell Res.*, **148**, 155.